# Insights on the Hypoglycemic Potential of *Crocus sativus* Tepal Polyphenols: An In Vitro and In Silico Study

**DOI:** 10.3390/ijms24119213

**Published:** 2023-05-24

**Authors:** Luisa Bellachioma, Camilla Morresi, Alfonso Albacete, Purificación A. Martínez-Melgarejo, Gianna Ferretti, Giorgia Giorgini, Roberta Galeazzi, Elisabetta Damiani, Tiziana Bacchetti

**Affiliations:** 1 Department of Life and Environmental Sciences, Marche Polytechnic University, Via Brecce Bianche, 60131 Ancona, Italy; luisabellachioma@gmail.com (L.B.); c.morresi@staff.univpm.it (C.M.); r.galeazzi@staff.univpm.it (R.G.); 2 Centro de Edafología y Biología Aplicada del Segura, Agencia Estatal Consejo Superior de Investigaciones Científicas (CEBAS-CSIC), Department of Plant Nutrition, Campus Universitario de Espinardo, E-30100 Murcia, Spain; alfonsoa.albacete@carm.es (A.A.); pmelgarejo@cebas.csic.es (P.A.M.-M.); 3 Department of Clinical Science and Odontostomatology, Marche Polytechnic University, Via Brecce Bianche, 60131 Ancona, Italy; g.ferretti@staff.univpm.it

**Keywords:** diabetes, polyphenols, post-prandial glycemia, intestinal glucose absorption, circular economy, *Crocus sativus*

## Abstract

Post-prandial hyperglycemia typical of diabetes mellitus could be alleviated using plant-derived compounds such as polyphenols, which could influence the activities of enzymes involved in carbohydrate digestion and of intestinal glucose transporters. Here, we report on the potential anti-hyperglycemic effect of *Crocus sativus* tepals compared to stigmas, within the framework of valorizing these by-products of the saffron industry, since the anti-diabetic properties of saffron are well-known, but not those of its tepals. In vitro assays showed that tepal extracts (TE) had a greater inhibitory action than stigma extracts (SE) on α-amylase activity (IC50: TE = 0.60 ± 0.09 mg/mL; SE = 1.10 ± 0.08 mg/mL; acarbose = 0.051 ± 0.07) and on glucose absorption in Caco-2 differentiated cells (TE = 1.20 ± 0.02 mg/mL; SE = 2.30 ± 0.02 mg/mL; phlorizin = 0.23 ± 0.01). Virtual screening performed with principal compounds from stigma and tepals of *C. sativus* and human pancreatic α-amylase, glucose transporter 2 (GLUT2) and sodium glucose co-transporter-1 (SGLT1) were validated via molecular docking, e.g., for human pancreatic α-amylase, epicatechin 3-o-gallate and catechin-3-o-gallate were the best scored ligands from tepals (−9.5 kcal/mol and −9.4 kcal/mol, respectively), while sesamin and episesamin were the best scored ones from stigmas (−10.1 kcal/mol). Overall, the results point to the potential of *C. sativus* tepal extracts in the prevention/management of diabetes, likely due to the rich pool of phytocompounds characterized using high-resolution mass spectrometry, some of which are capable of binding and interacting with proteins involved in starch digestion and intestinal glucose transport.

## 1. Introduction

Diabetes mellitus is a common chronic disease characterized by a persistent hyperglycemic condition due to a dysfunction in pancreatic insulin production (typical of type 1 diabetes: T1D) or to a reduction in insulin activity in peripheral tissues, known as insulin resistance (typical of type 2 diabetes: T2D). According to the International Diabetes Foundation, in 2022, over 537 million people worldwide live with this disease and this number is expected to grow, especially in low and middle-income countries [1]. T2D is the most prevalent, and its onset is influenced by several factors such as genetics, age, gender, microbiota, lifestyle and obesity. One beneficial approach, amongst the several available in the management of diabetes [2], is to reduce post-prandial hyperglycemia through preventing dietary carbohydrate digestion and absorption. In fact, high post-prandial plasma glucose concentrations and repeated blood glucose spikes are associated with an increased risk for insulin resistance, which lead to the development of T2D and metabolic syndrome [3]. Indeed, some of the commercially active antagonists for diabetes management, such as acarbose [4], inhibit carbohydrate-hydrolyzing enzymes such as α-amylase and α-glucosidase. Their inhibition prevents starch hydrolysis in the intestine, thus contributing to lower postprandial glucose levels through decreasing glucose absorption [4]. However, the only three carbohydrate-hydrolyzing enzyme inhibitors currently used in clinical practice, namely acarbose, miglitol and voglibose, are not without issues and side effects [5]. Drawbacks include stomach distress, diarrhea, bloating and nausea [5]. Intestinal glucose transporters such as SGLT1 and GLUT2 play a role in glucose homeostasis and therefore they also represent useful targets for the management of diabetes [6].

Since medicinal plants and traditional treatments have been used throughout history as a remedy for all sorts of medical disorders including diabetes, it comes without surprise that exploring natural inhibitors of enzymes involved in carbohydrate digestion and of intestinal glucose transporters as potential combinatorial therapeutics is appealing for the management/prevention of post-prandial hyperglycemia [7,8,9]. In fact, there is a wealth of evidence from in vivo and in vitro studies showing the potential of plant-derived molecules such as polyphenols (flavonols, catechins, theaflavins and tannins) from vegetables, fruits, mushrooms, oils and spices to influence the activities of amylolytic enzymes [7,8,10,11,12,13,14,15]. Polyphenols have been reported to inhibit pancreatic α-amylase and intestinal β-glucosidase through binding to the pockets of these enzymes, forming enzyme-inhibitor complexes, and a relationship between the structural characteristics of polyphenols and their α-amylase inhibiting properties exists [16,17].

*Crocus sativus*, known as saffron, is a plant belonging to the *Iridaceae* family whose use in diabetes has been known since ancient times [18,19]. Stigmas are generally considered as the most valuable part of saffron. However, the dried stigmas represent only 7.4% of *C. sativus* flowers and large amounts of floral bio-residues, such as tepals, are generated and wasted in the production of saffron. We recently focused our attention on valorizing this waste product through studying hot and cold herbal infusions of the flower parts and noted that tepals, via an untargeted phenolic profiling, are a rich source of polyphenols and that their content in polyphenols and flavonoids is higher compared to stigmas [20,21]. The present study aimed to investigate in vitro the potential anti-hyperglycemic effect of *Crocus sativus* tepals compared to stigmas, within the framework of valorizing these by-products of the saffron industry. Indeed, a wealth of in vitro and in vivo studies as well as clinical trials indicate that saffron and its constituents have antidiabetic effects, recently reviewed in [18,19]. However, there are very limited studies concerning the hypoglycemic properties of tepals. Menghini et al. studied in vitro the effects of anther and tepal extract and of stigma extract from *C. sativus* on the activity of α-amylase [22]. The results revealed that anther and tepal extract was the most effective in inhibiting α-amylase activity. However, this study did not report any data on tepal extracts without anthers nor data explaining the molecular interaction mechanisms. Wali et al. investigated the ability of organic solvent extracts of *C. sativus* tepals but not aqueous extracts to inhibit α-glucosidase, whereas the effects on α-amylase were not explored [23]. Instead, Ouahhoud et al. confirmed the anti-diabetic properties of tepals in an in vivo study on diabetic rats; they reported that administration of the hydroalcoholic extract of tepals in these rats significantly decreased body weight and reduced blood glucose, plasma triglycerides, cholesterol, urea, creatinine, aspartate amino transferase (AST) and alanine amino transferase (ALT) levels compared to untreated diabetic rats [24]. Amraei et al. also conducted a similar in vivo study on diabetic Wistar rats and found that 100–300 mg/kg tepal extracts were capable of influencing high serum levels of glucose, glycated hemoglobin (HbA1c) and insulin, similar to the effects induced by metformin and glibenclamide treatment [25].

However, information on the effects of tepal extract (TE) compared to stigma extract (SE) on glucose intestinal absorption and on expression of intestinal glucose transporters such as SGLT1 and GLUT2 is lacking. This information is nevertheless important considering that the intestinal barrier is primarily the first one that TEs and SEs would come in contact with once ingested. In fact, polyphenols are scarcely bioavailable [26]; hence, one would expect that their main effect would be exerted at the level of the intestine. Moreover, the intestine is the target site of antidiabetic drugs (inhibitors of enzymes and glucose receptors). To fill this knowledge gap, we relied on the experimental approach of several literature studies that define the potential hypoglycemic properties of plant extracts [27,28,29,30,31] using a differentiated intestinal human colon adenocarcinoma cell line (Caco-2) and with the support of a computational study. The inhibitory action of stigma and tepal extracts on α-amylase activity was determined using an in vitro assay, and their effects were compared to that of the pharmacological inhibitor, acarbose. Instead, their effects on glucose transporters were evaluated in vitro on Caco-2 cells. Further insights on the potential mechanisms that could explain how the principal phytocompounds found in these flower parts modulate the activity of enzymes involved in carbohydrate digestion and glucose transporters were obtained for the first time via in silico docking studies.

## 2. Results and Discussion

### 2.1. Biochemical Characterization of Tepal and Stigma Extracts

The biochemical characterization of the extracts shows that higher levels of total polyphenols (TPC) and total flavonoids (TFC) are present in tepal extract (TE) compared to stigma extract (SE) (*p* < 0.001) (Table 1). Furthermore, TE exhibited a higher antioxidant capacity as evaluated using the oxygen radical absorbance capacity (ORAC) assay (*p* < 0.001) (Table 1), which reflects the higher content of flavonoids, the major class of polyphenols, well-known to act as antioxidants both in vitro and in vivo [32]. The data obtained are consistent with those from our previous studies which showed that TE had higher antibacterial activity than SE, which was correlated with its higher total polyphenol content [20,21]. Further support also comes from our earlier study where hot infusions prepared with tepals and stigmas of *C. sativus* were compared, which showed that tepal infusions scored higher in terms of antioxidant capacity and TPC compared to stigma infusions [21]. The levels of TPC found in TE and SE were shown to be either higher or lower compared to those found in the literature depending on the solvents and extraction techniques used and the origin of the cultivars [33]. For example, using conventional maceration, Lakka et al. found a three-fold higher TPC in water extracts of tepals; however, they used a ten-fold higher tepal:water ratio than ours [34]. Stelluti et al. instead found three-fold lower values of TPC in tepal water extracts than ours despite a four-fold higher tepal:water ratio but less extraction time [35].

Since the focus of the present study is on the valorization of tepals that are discarded in the saffron industry, the detailed composition of the tepal extracts obtained after purification using solid phase extraction (SPE) of the water extract was characterized via ultra-high performance liquid chromatography coupled to high-resolution mass spectrometry (U-HPLC-HRMS). A total of 96 phenolic compounds were putatively annotated to include flavonoids (i.e., anthocyanins, dihydrochalcones, flavonols, isoflavonoids), phenolic acids (hydroxybenzoic acids, hydroxycinnamic acids, hydroxyphenypropanoic acids), stilbenes and other polyphenols. The detailed list containing all the phenolic compounds annotated is reported in the Supplementary Table S1, together with their abundance. In agreement with our previous studies, and with those of other authors, the phenolic profile of the tepal infusion was mainly represented by flavonoids, with anthocyanins being the most abundant subclass [20,21,22,36,37,38].

### 2.2. Inhibition of α-Amylase Activity Using Extracts from Stigma and Tepal

An important strategy for managing post-prandial glycemic levels and diabetes care control is through α-amylase inhibition since it is the main digestive enzyme involved in the hydrolysis of starch. Isolated α-amylase was incubated with increasing concentrations of TE and SE and activity data in the presence of increasing concentrations of extracts were expressed as the percentage of uninhibited α-amylase activity. As a “positive” control, acarbose was used in parallel incubations. TE and SE showed a dose-dependent inhibitory activity of α-amylase and since IC50 (half maximal inhibitory concentration) is considered as an acceptable parameter describing the inhibitory activity of an inhibitor, IC50 values were calculated from the enzyme activity data (Supplementary Figure S1), to compare the inhibition induced by the two extracts. As shown in Table 2, TE exhibited a lower IC50 than SE, suggesting the higher inhibitory activity of the former. The degree of inhibition of enzymatic activity could be related to the higher amount and different composition of polyphenolic compounds present in TE compared with SE. In fact, the 50% inhibition of α-amylase activity was observed using TE and SE containing similar levels of total polyphenols (Table 2) but requiring almost twice the amount of SE compared to TE (1.10 vs. 0.6 mg/mL, respectively). These results are in agreement with previous studies recently reviewed by Corkovic et al. in 2022 [39], where dietary polyphenols have shown potential inhibitory activity against α-amylase. The IC50 values of TE and SE are similar to those reported by other authors for different polyphenolic rich extracts such as mulberry fruit (*Morus alba*) extract (IC50 = 0.30 mg/mL) [40] or encapsulated grape skin phenolics (IC50 = 0.44 mg/mL) [41], olive pomace (IC50 = 0.56 mg/mL) [42] or propolis extract (IC50 = 0.55 mg/mL) [43]. It has been now well accepted that α-amylase inhibition by polyphenols is caused in most cases by their direct interactions [44,45,46]. This inhibitory activity of polyphenols against α-amylase is highly related to their phenolic chemical structure [17], although structure-inhibition relationships of polyphenols present in *C. sativus* floral parts have not been studied. Therefore, to gain further insights on the interactions between polyphenols contained in TE and SE with α-amylase, which could explain their inhibitory activity, a computational study was performed, as reported in Section 2.3.

### 2.3. Inhibition of Glucose Transport Using Extracts from Stigmas and Tepals

After starch digestion in the small intestine by digestive enzymes, the resulting glucose in the bowel lumen is absorbed by enterocytes and transported in the bloodstream under the action of glucose transporters, such as SGLT1. Another transporter, GLUT2 has been detected at both the apical and basolateral membrane of Caco-2 cells [47].

Therefore, the effect of TE and SE on glucose absorption and on the levels of SGLT1 and GLUT 2 were investigated using Caco-2 differentiated cells as a model of the intestinal epithelial barrier. Phlorizin, a polyphenol and well-known inhibitor of SGLT1, was used as a reference inhibitor. Addition of TE and SE to the apical side of Caco-2 cells led to a dose-dependent inhibition of the basolateral transport of glucose after 6 h, while at 2 h of incubation, no significant decreases were observed. From the dose-dependent curves, the concentration required for 50% inhibition of glucose (IC50) was calculated (Supplementary Figure S2). These IC50 values show the capacity of both extracts to inhibit glucose transport, with a lower IC50 for TE compared to SE, suggesting a higher inhibitory action of the former (Table 3). The data indicate that TE and SE may interfere with the transport function of SGLT1 and GLUT2, resulting in decreased glucose uptake into Caco-2 cells. Indeed, polyphenols have been reported to reduce glucose uptake through direct inhibition of SGLT1 and GLUT2 in Caco-2 cells [48].

**Figure 1:**
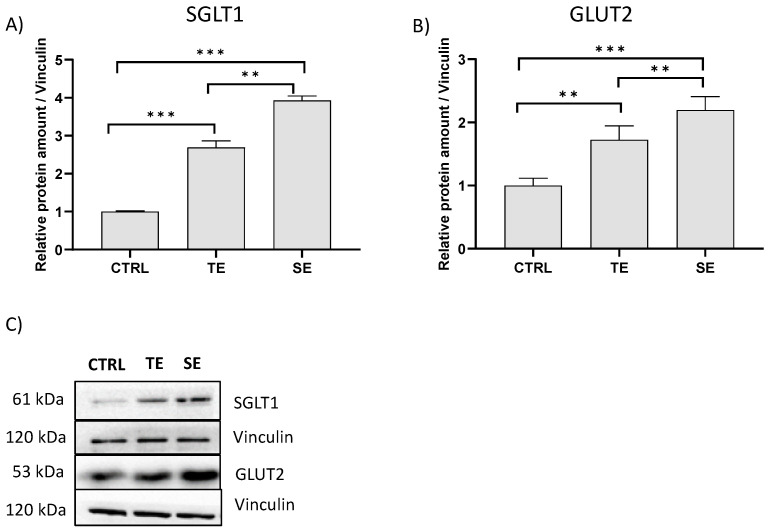
Effect of tepal (TE) and stigma (SE) extracts on the levels of glucose transporters in differentiated Caco-2 cells. Densitometric analysis of SGLT1 (**A**) and GLUT2 (**B**) transporters and representative Western blots (**C**). Differentiated Caco-2 cells were incubated for 6 h in the presence of 5 mM glucose (CTRL) and with TE or SE (50 μg GAE/mL). Densitometric data are normalized to vinculin expression levels. Data are presented as the mean ± S.E.M. (*n* = 3). ** *p* < 0.01; *** *p* < 0.001.

Therefore, to further determine whether the reduced rate of glucose transport caused by TE and SE is associated with alterations in membrane glucose transporters, the effect of the two extracts on protein expression of SGLT1 and GLUT2 in Caco-2 differentiated cells was investigated via immunoblotting (Figure 1). As shown in Figure 1, treatment for 6 h with TE and SE (50 μg GAE/mL) in the presence of glucose caused an unexpected significant increase in both SGLT1 and GLUT2 protein levels with respect to control cells. This result may be ascribed to the inhibition of glucose absorption by TE and SE polyphenols that could lead to fuel starvation in cells, inducing them to upregulate glucose transporters, as already observed for tea catechins in Caco-2 cells [49]. However, several literature reports indicate that glucose transporters are downregulated by polyphenol extracts and several mechanisms have been delineated. In general, polyphenols appear to suppress the expression of glucose transporters through stimulating the AMPK/SGLT1 pathway [50,51,52], or other intracellular pathways, including the Na^+^, K^+^-ATPase/SGLT1 cascade [53]. Theaflavins, for example, were shown to inhibit glucose transport across Caco-2 cell monolayers through the suppression of SGLT1 expression, partly via the activation of the intracellular Ca^2+^/CaMKK β/AMPK signaling pathway [54].

The evaluation of transepithelial electrical resistance (TEER) across the monolayer of Caco-2 differentiated cells was also used to assess the effect of the treatments on the integrity of the intestinal barrier [55]. No significant modifications in TEER across Caco-2 cells’ monolayer were observed in cells treated with TE and SE under our experimental conditions. This result suggests that the floral extracts from *C. sativus* exert an inhibitory effect on glucose absorption without modifying the integrity of the cell monolayer (Supplementary Figure S3).

Based on the above results, the interactions between polyphenols contained in TE and SE and the glucose transporters SGLT1 and GLUT 2 were elucidated using a computational approach, to provide a better understanding of the possible mechanisms involved.

### 2.4. Results of In Silico Studies on α-Amylase

The α-amylase aminoacidic sequence is conserved among the mammalian class and a huge number of computational studies have been carried out on this enzyme complexed with its inhibitors [56,57], resulting in many retrievable crystallographic structures (i.e., porcine α-amylase with acarbose (pdb code 1OSE) and the human α-amylase with acarviostatins (pdb codes 3OLE, 3OLD, 3OLG, 3OLI)).

The enzyme structure Is organized into three domains (A,B,C): the A domain is consists of a TIM barrel (α/β) supersecondary structure enclosing the active site with the cofactor ion Cl^−^_,_ which is further divided into different subsites on the basis of substrates’ and inhibitors’ binding modes, and its electrostatic potential is prevalently negative; the B domain includes the Ca^2+^ ion relevant for the maintenance of the enzyme structure; the C domain represents the carbohydrate binding domain (CBD).

#### 2.4.1. Structure Analysis and Binding Site Mapping

The focus of this in silico study was to evaluate the inhibitory effect of compounds extracted from tepals and stigma of *C. sativus*, as lead compounds interacting with the catalytic site of the human α-amylase enzyme.

**Figure 2:**
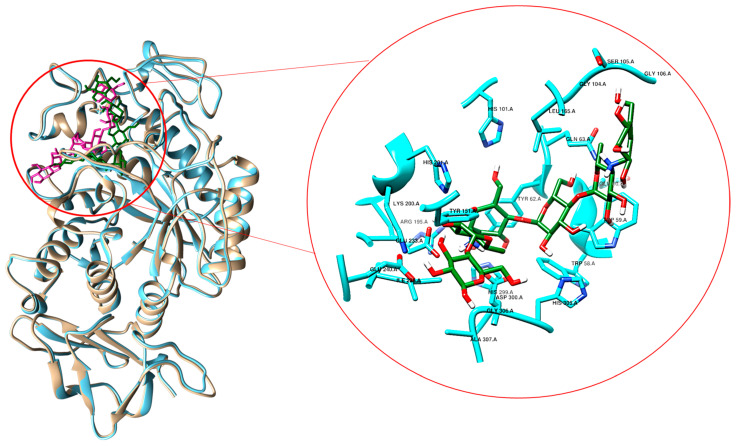
Superimposition of porcine α-amylase and human α-amylase in complex with acarviostatin (in violet, 3OLI), acarbose (in green, 1OSE); in the focus section, principal residues (cyan) interacting with acarbose (green) are shown.

Before initiating the virtual screening process, the glucose binding site was mapped based on the X-ray structure of human α-amylase in complex with acarviostatin (pdb code 3OLI), porcine α-amylase with acarbose (pdb code 1OSE) and pig pancreatic α-amylase with glucose (pdb code 1PPI). These X-ray-solved structures were superimposed to confirm the structural similarity, and their sequences were also aligned for searching matching residues. The superimposition also confirmed the positioning of both the inhibitors and glucose in the same binding cleft, thus validating the binding site mapping (Figure 2). Sequences are homologue and express 92% of identity. In particular, residues within the active site are well conserved except for the residues T vs. V163. The amino acids of the catalytic triad are D197, E233 and D300.

#### 2.4.2. Virtual Screening (HTVS) with Stigma and Tepal Compounds against α-Amylase

**Figure 3:**
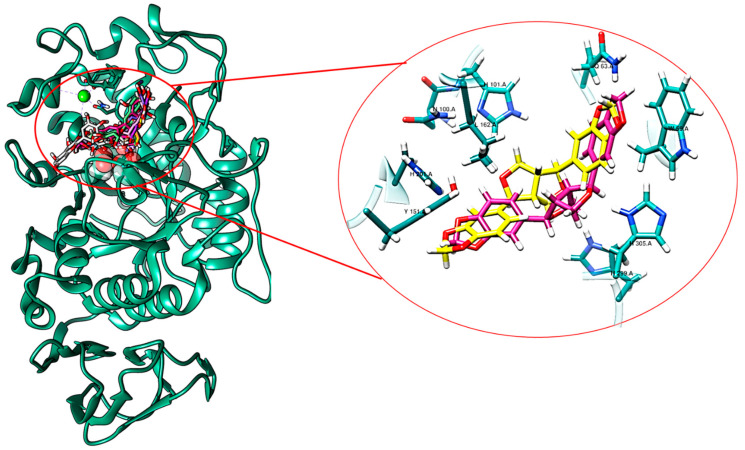
Computational prediction of the two best-scoring compounds: episesamin (violet tubes) and sesamin (yellow tubes) binding modes; in green sphere, Cl^−^ ion as cofactor is also shown. They are visualized superimposed inside the α-amylase binding pocket (aquamarine ribbons representation). In the focus, a close-up of the main interactions with the cleft residues (cyan tubes) for the same two best-scoring compounds present in *C. sativus* stigmas, episesamin (in violet) and sesamin (in yellow), are shown.

**Figure 4:**
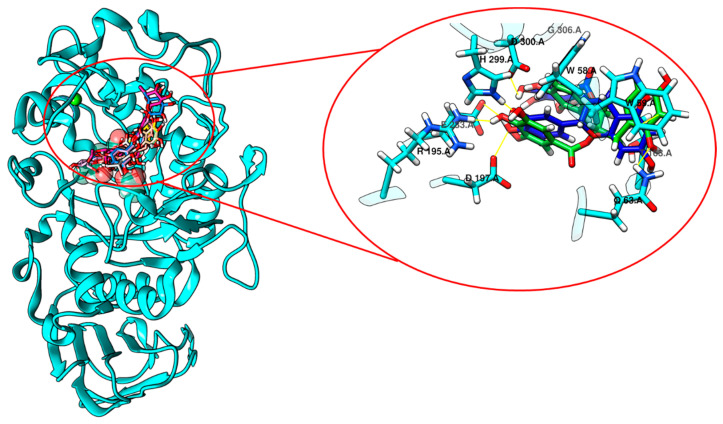
Computational prediction of the two best-scoring compounds present in *C. sativus* tepals: cathechin-3-o-gallate (green tubes) and epicathechin-3-o-gallate (blue tubes); in green, CPK sphere Cl^−^ ion as cofactor and in red, CPK spheres Ca^2+^ ions (responsible for supersecondary structures) are also shown. They are visualized superimposed inside the α-amylase (cyan ribbons) binding pocket. In the focus, a close-up of the main interactions with the cleft residues (cyan tubes) for the same two best-scoring compounds, cathechin-3-o-gallate (green tubes) and epicathechin-3-o-gallate (blue tubes), are shown.

The virtual screening performed with compounds from stigma and tepals of *C. sativus* and human pancreatic α-amylase (3OLI) led to interesting results. The best-scoring compounds in terms of binding energies and positioning after HTVS are reported in Table 4 and Figure 3 for stigmas and Table 5 and Figure 4 for tepals. It is worth noting that all the best-scoring compounds occupy the same binding site identified for acarbose and acarviostatin in the X-ray structures.

All these selected phytocompounds from stigmas establish relevant interactions with residues of the catalytic triad, namely H-bonding and π-stacking, with the following amino acids: W59, Y62, Q63, L165, E233, H299 and H305. Among the polyphenolic compounds, lignans are also present and contribute to inhibition. Indeed, polyphenolic compounds are known as α-amylase inhibitors and many experimental and computational studies are reported in the literature pointing out that the hydroxylic and aromatic moieties are directly involved in the interactions with the residues of the catalytic triad [58,59,60]. The binding range affinity is −10.1/−8.80 kcal/mol (Table 4).

The same behavior was observed for the compounds extracted from tepals. In Table 5, the best polyphenolic compounds together with their binding affinities, ranging from −9.9 kcal/mol to −8.9 kcal/mol, are reported. Both stigma and tepal compounds establish π interactions and H-bonds with amino acids in the inhibition pocket: W58, R195, D197, K200, E240, E233, H299, D300, G306, H305 and H308. It is worth noting that for both stigma and tepal best-scoring compounds, the binding affinities are higher than the acarbose one within the porcine α-amylase. This is particularly important since acarbose is one of the best inhibitors described in the literature [61,62]. In light of these obtained results, the selected phytochemicals (Table 4 and Table 5) could act as competitive inhibitors within the catalytic site of α-amylase, thus hindering glucose binding.

Furthermore, in order to test the binding strength, two systems, α-amylase with catechin-3-o-gallate and α-amylase with episesamin, a flavonol and lignan compound, respectively, underwent molecular dynamics simulations. These ligands were chosen as they were the best-scoring compounds from the molecular docking, and considering their dynamical behavior, we confirmed their binding stability. As a result, along the 50 ns trajectory, the binding positioning was confirmed with catechin-3-o-gallate, resulting in the most stable one. Therefore, a competitive mechanism can be hypothesized, since these two ligands (episesamin and epicatechin-3-o-gallate) occupy the same pocket as acarbose and acarviostatin, two well-known characterized inhibitors.

#### 2.4.3. Structural Investigation in GLUT2 Binding Site

**Figure 5:**
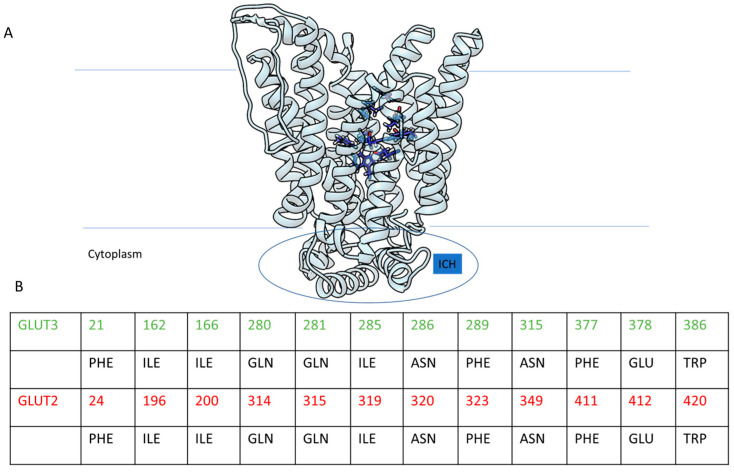
(**A**) GLUT2 receptor (light cyan) with the glucose binding site residues highlighted in blue tubes and oriented in the membrane, thus showing the ICH domain inside the cytoplasm; (**B**) number and residues correlation between aminoacidic composition of the binding pocket in GLUT2 (red in the table) and GLUT3 (green in the table).

The receptor GLUT2 embedded within the enterocyte membrane can be present in an outward and inward conformation in accordance with its activity of glucose transporter from the intestinal lumen into the cell. The identification of the glucose binding site within the human GLUT2 receptor in its outward conformation (Figure 5A) was achieved considering the site mapped within the X-ray-solved structure of the human receptor glucose transporter 3 (GLUT3) (pdb codes: 4ZWC-4ZW9) [63]. The GLUT2 binding site was detected through superimposing GLUT2 and GLUT3 structures and evaluating their aminoacidic sequences (Table S2). The aminoacidic sequences in the binding site are well conserved but the numbering changes since the two full sequences are not identical, although they show a high percentage of similarity equal to 53.62% (Figure 5B).

#### 2.4.4. Virtual Screening of Stigma and Tepals Compounds with GLUT2 Receptor

**Figure 6:**
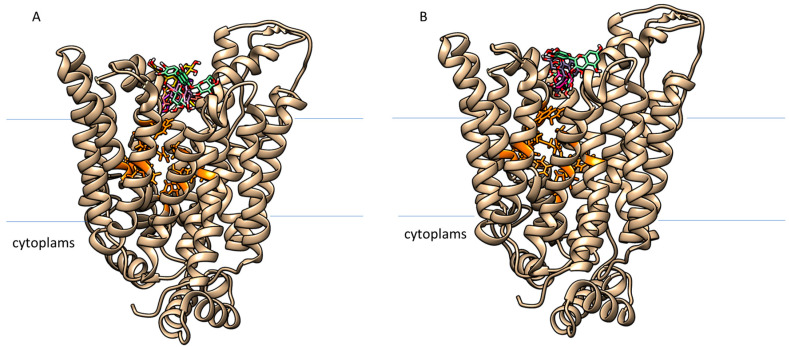
Computational prediction of the docked poses of the best-scoring compounds (in colored tubes) extracted from stigmas (**A**) (Table 6) (Episesamin in salmon; Cyanidin-3,5-o-diglucoside in watergreen; Carnosol in violet; Delphinidin-3,5-o-diglucoside in yellow and Cyanidin in pink tubes) and tepals (**B**) (Table 7) (Delphinidin-3-o-glucosylglucoside in watergreen; Quercetin-3-o-acetylrhamnoside violet; Epirosmanol in purple tubes) of *C. sativus* for GLUT2 receptor (gold representation in the image); all the chosen compounds after the virtual screening process are located at the upper site cleft of the GLUT2 receptor. The residues involved in glucose binding are also shown as orange tubes to better locate the relative position of the two binding clefts.

As for α-amylase, HTVS with both libraries (stigmas and tepals) was carried out against GLUT2. The obtained binding energies for both screened libraries range from −8.60 kcal/mol to −8.0 kcal/mol and in both cases, two different binding sites can be identified: one corresponding to the glucose site, and the other located at the upper site of the receptor in the extracellular region (just along the glucose entrance pathway) (Figure 6, Table 6 and Table 7).

From the results obtained, a non-competitive inhibition may be hypothesized since the selected phytocompounds occupy the upper domain of the receptor, at the interface with the extracellular domain on the apical side of enterocytes facing the intestinal lumen. This zone corresponds to the entrance pathway for glucose, whose binding cleft is buried inside the receptor. In view of these results, this non-competitive inhibitory mechanism induced by the polyphenols may block the sugar’s access, thereby preventing its binding. This finding is also supported by data in the literature on the flavonoid quercetin, reported to have a similar inhibitory mechanism [64].

#### 2.4.5. Structural Investigation of the Binding Site for the Glucose Transporter SGLT1

**Figure 7:**
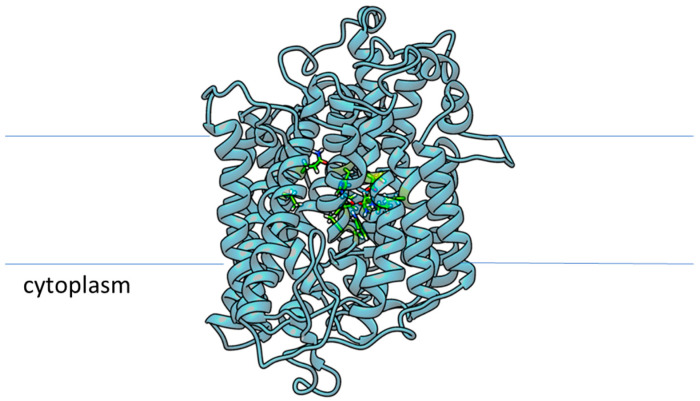
Structural representation of h-SGLT1 receptor (cornflower blue ribbons) in its membrane orientation; the mapped residues in the central cavity corresponding to the glucose binding cleft are highlighted as green tubes.

In order to evaluate the possible inhibitory effect on human SGLT1 glucose transporter from *C. sativus* phytocompounds present in the two tepal and stigma libraries, the cryo-EM-solved structure of this receptor was retrieved (pdb code: 7SL8) [65]. On this structure, an in silico study was carried out to better define the glucose binding site [65], and for the reported results, both the binding pocket mapping and mechanism of the SGLT1 family of glucose transport were reconstructed (Figure 7). The principal binding cleft residues are the following: K321, E102, N78, Y290, Q457, S460, H83, A287 and W291.

**Figure 8:**
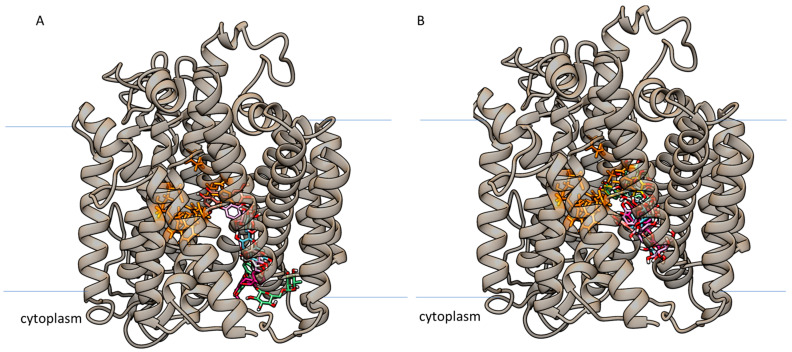
h-SGLT1 glucose transporter with the docked poses of the best-scoring compounds (colored tubes) extracted from stigmas (**A**) and tepals (**B**) of *C. sativus* after the virtual screening process. The binding cleft is positioned below the glucose binding cleft, and the residues involved in this glucose binding cleft are shown in orange.

The virtual screening results show high affinities for the ligands reported in Table 8 and Table 9, but their positioning is not exactly within the glucose binding pocket as reported in the literature for the h-SGLT1 inhibitor LX2671 [66]. In fact, the identified binding pocket for the best-scoring stigma and tepal compounds falls close to the glucose binding site. However, this site is located below the glucose one at the interface with the cytoplasm (Figure 8). It is worth noting that on the basis of the ligands’ position, it would be reasonable to expect that these compounds may not prevent glucose binding, but they can hinder glucose’s release and absorption from its binding pocket. Therefore, as shown for GLUT2, a non-competitive inhibition can be assumed.

## 3. Materials and Methods

### 3.1. Plant Material and Extracts Preparation

The plant material was kindly donated by local farms. *Crocus sativus* flowers were collected from the farms “Tesoro delle Api” (Fermo, Italy) and “Stachys” (Recanati, MC, Italy) in October–November 2022. The stigmas were immediately and carefully manually separated from the tepals in the laboratory. About 800 g flowers were obtained representing 80% tepals and 7% stigmas, in agreement with previous studies [22].

Tepals and stigmas were initially frozen at −20 °C before being lyophilized in a freeze dryer (LYOQUEST-55, Seneco, Milano, Italy). The yield of lyophilized material starting from fresh material was 15% for tepals and 20% for stigmas. The freeze-dried samples were weighed, ground and then vacuum-packed and stored at room temperature in the dark. Briefly, freeze-dried tepals and stigmas (0.5 g each) were extracted in 100 mL double-distilled water following the method of Rodriguez-Ruiz et al. (2016) [67]. The extracts were left on a laboratory shaker overnight at 4 °C and centrifuged twice at 2000× *g* for 10 min. Finally, the collected supernatants were filtered through 0.45 μm Whatman filter paper membranes. Furthermore, water extracts were further purified at once using solid-phase extraction (SPE), a rapid, simple and economical technique. SPE allows the extraction of phenolics and the removal of sugars and other highly polar compounds (e.g., organic acids, amino acids, proteins). This was performed using SPE Chromabond-PA columns (3 mL/500 mg) purchased from Carlo Erba (Milano, Italy) according to the protocol reported by Saeidi et al. [68]. Tepal and stigma extracts (5 mL) were mixed with 25 mL of double-distilled deionized water and adjusted to pH 3 with HCl. The SPE polyamide cartridge was activated with 5 mL of n-hexane, 5 mL of methanol and conditioned using 10 mL of double-distilled deionized water. The extracts were passed through the cartridge and washed with 8 mL water/methanol (90:10 *v*/*v*, adjusted to pH 3 with HCl) to remove interferences and eluted with 4 mL HPLC-grade methanol. The alcoholic fraction was removed via SpeedVac. The dried extracts were then resuspended in 200 μL of double distilled water and stored at −20 °C.

### 3.2. Total Polyphenols and Flavonoids Content

Polyphenolic quantification (TPC) of the tepal and stigma extracts was first carried out in order to use standardized amounts of extracts in subsequent analyses. The Folin–Ciocalteu method was used as previously described by Singleton et al. [69]. Briefly, 20 µL of each extract (diluted 5 times with double-distilled water) was mixed with 100 μL of Folin–Ciocalteu reagent and 300 µL of Na_2_CO_3_ (20% *w*/*v*) and incubated for 30 min at 40 °C in the dark. The absorbance was then read at 765 nm in a 96-well microplate reader (Synergy-HT, BioTek, Winooski, VT, USA) and the results expressed as mg gallic acid equivalents (GAE) per g of dry weight (mg GAE/g), since gallic acid was used to create the standard curve. Polyphenol yield of SPE was evaluated as a balance between TPC initially found in water extract and those in the retained and unretained fraction after SPE. The results demonstrated that polyphenols in purified polyphenolic extract accounted for about 80% of those in the water extract [70].

Total flavonoid content was measured using a colorimetric assay according to previous studies [20]. Briefly, 0.5 mL appropriately diluted extract, (+)-catechin standard solution, or water as blank were mixed with 5% NaNO_2_ (0.150 mL) followed by 10% AlCl_3_ (0.150 mL). Samples were incubated for 15 min at room temperature in the dark and the absorbance was read at 415 nm in a 96-well microplate reader (Synergy-HT, BioTek, Winooski, VT, USA). The results are expressed as mg of catechin equivalent (CE) per g of dry weight (mg CE/g).

### 3.3. Antioxidant Capacity

Antioxidant capacity was measured using the ORAC assay according to Gillespie et al. [71]. Briefly, 25 µL of the diluted sample, blank or Trolox calibration solutions were mixed with 0.150 mL of 0.08 µM fluorescein and incubated for 15 min at 37 °C. Subsequently, 25 µL of 2,2′-azobis(2-amidinopropane) dihydrochloride (AAPH) solution (150 mM) was added as a peroxyl radical generator. The fluorescence was measured every 2 min for 4 h using fluorescence filters for an excitation wavelength of 485 nm and an emission wavelength of 530 nm in a 96-well microplate reader (Synergy-HT, BioTek, Winooski, VT, USA). The final ORAC values were calculated using the net area under the decay curves and are expressed as millimoles of Trolox equivalents per g of dry weight (mmol Trolox eq./g).

### 3.4. Metabolomic Profiling

Tepal extracts were filtered through 13 mm diameter Millex filters with 0.22 µm pore size nylon membrane (Millipore, Bedford, MA, USA). Ten microliters of filtered extract were injected using a ultra-high performance liquid chromatography device (UHPLC, Accela Series, ThermoFisher Scientific, Waltham, MA, USA) coupled to a high-resolution mass spectrometer (Exactive, ThermoFisher Scientific, Waltham, MA, USA) consisting of an Orbitrap detector and using a heated electrospray ionization (HESI) interface. Data processing was carried out through the Xcalibur software (version 4.3, ThermoFisher Scientific, Waltham, MA, USA); the XCMS metabolomics platform (Scripps Center for Metabolomics and Mass Spectrometry, La Jolla, CA, USA) and the KEGG, PUBCHEM and PHENOL-EXPLORER chemical databases, among others. For fine-tuning the analysis method, the molecular formulas of the compounds were searched in the PUBCHEM platform and entered in the Qual Browser package of the Xcalibur software, where mass/charge (*m*/*z*) ratios of each metabolite were identified in the negative mode, adjusting a mass tolerance of ≤2 ppm in the Processing Setup Package. Additionally, correlations between compounds of the same metabolic pathway and the LogP coefficient were used to accurately identify these metabolites.

### 3.5. α-Amylase Inhibition

The tepal and stigma extracts were assayed for their anti-diabetic activities through analyzing their inhibitory effect on α-amylase according to the method reported by Kim et al. 2000 [72]. Briefly, tepal and stigma extracts (0–200 μg GAE/mL) or acarbose (0–100 μg/mL) were incubated with 100 μL of porcine amylase (2 U/mL in 10 mM phosphate buffer, pH 6.8) for 10 min at 37 °C. The substrate, blocked p-nitrophenyl-α-d-maltoheptaoside (BpNPG7) (3.7 mM) dissolved in DPBS, was then added. The kinetics of the reaction was monitored at 37 °C for 10 min. The final absorbances were measured at 405 nm in a 96-well microplate reader (Synergy-HT, BioTek, Winooski, VT, USA). Three replicates were carried out for each sample. The results are expressed as percentage (%) of inhibition using the following formula:

% inhibition = 100 × (A_0_ − A_s_)/A_0_

where A_0_ is the absorbance of the control and A_s_ is the absorbance of the sample containing the extracts, and they were used to construct the dose-dependent curves from which the IC50 values were obtained.

### 3.6. Cell Cultures

Human colorectal adenocarcinoma cells, Caco-2, were purchased from the American Type Culture Collection (Rockville, MD, USA) and cultured at 37 °C, 5% CO_2_ in a humidified atmosphere. Cells were seeded at a density of 1 × 10^5^ cells/well in 12-well Transwell plates (12 mm, with 0.4 µm pore polycarbonate membrane insert, Corning, NY, USA) and differentiated for 21 days in Dulbecco’s Modified Eagle’s Medium (DMEM) with 10% FBS. The medium was replaced every 2 days. The integrity of each cell monolayer was routinely checked via the trans-epithelial electrical resistance (TEER) technique using an EVOM chopstick electrode (Millicell ERS-2, EMD Millipore, Billerica, MA, USA).

#### 3.6.1. Evaluation of Tepal and Stigma Extracts on Glucose Uptake

The glucose absorption assay was carried out as previously reported by Morresi et al. [29]. Differentiated cells were washed twice in DPBS. Increasing concentrations of tepal and stigma extracts (0–150 μg GAE/mL) (prepared in DPBS) were each separately co-incubated in the apical compartment of the Transwell plate with 5 mM glucose for 6 h. Phlorizin (0–500 μg/mL) was used as the positive control. In the basolateral compartment of the Transwell plate, only DPBS was added. Cells were incubated at 37 °C in a humidified atmosphere and 5% CO_2_ for the duration of the experiment. At different time intervals (0, 2 and 6 h), aliquots of the medium from the basolateral side were collected, and glucose concentration was analyzed using 4-hydroxybenzhydrazide (PAHBAH) according to the method of Moretti et al., 2008 [73]. The collected samples (10 µL) were added to 65.8 mM PAHBAH reagent at a volume ratio of 1:30 in test tubes. The mixtures were then incubated for 10 min at 90 °C in a thermostatic bath. After incubation, the samples were loaded onto a 96-well plate and read in a microplate reader at 410 nm. The analyses were performed in triplicate and the results are expressed as percentage (%) of inhibition of glucose passage compared to the control (CTRL). Glucose (0.01–5 mM) was used to create the standard curve.

#### 3.6.2. Evaluation of Tepal and Stigma Extracts on Gut Permeability

The integrity of the monolayer was evaluated using TEER measurements [29]. Resistance measurement was performed using TEER. Differentiated cells were incubated for different times (0, 2 and 6 h) in the presence or absence of tepal and stigma extracts (0–150 μg GAE/mL). TEER was measured using an EVOM with a chopstick electrode (Millicell ERS-2, EMD Millipore, Billerica, MA, USA). The electrode was immersed at a 90° angle with one tip in the basolateral chamber and the other in the apical chamber. Care was taken to avoid electrode contact with the monolayer and triplicate measurements were recorded for each monolayer. An insert without cells was used as a blank and its mean resistance was subtracted from all samples. These experiments were performed in triplicate.

#### 3.6.3. Expression of the Glucose Transporters

Expression of glucose transporters in differentiated Caco-2 cells was carried out via immunoblotting. Cells were incubated for 6 h in the absence or in the presence of tepal and stigma extracts (50 μg GAE/mL); cells were then harvested, washed twice with DPBS and the cell pellet was solubilized in 150 μL RIPA buffer. Protein concentration of cell lysates was determined using the Bicinchoninic Acid (BCA) Assay with bovine albumin as standard [74]. Proteins (25 μg) were mixed with loading buffer, denatured at 100 °C for 5 min, loaded on 12% polyacrylamide gels and subjected to SDS-PAGE. After electrophoresis, proteins were transferred onto polyvinylidene fluoride (PVDF) membranes at 4 °C. After regular blocking with EveryBlot Blocking Buffer for 5 min and washing with Tris-buffered saline 0.1% Tween 20, the membranes were incubated overnight with primary antibodies at 4 °C. For the determination of glucose receptors, anti-GLUT2 antibody (JJ20-21 Invitrogen, Waltham, MA, USA, diluted 1:500) and anti-SGLT1 antibody (Pa5-84085 Invitrogen, USA, diluted 1:200) were used, while vinculin was used as housekeeping protein (sc-25336 Santa Cruz Biotechnology, Dallas, TX, USA, diluted 1:200). Anti-mouse (sc-2005 Santa Cruz Biotechnology, Dallas, TX, USA, diluted 1:100,000) and anti-rabbit (12–348 Sigma-Aldrich, Darmstadt, Germany, diluted 1:150,000) were used as secondary antibodies. Images were acquired on a ChemiDoc XRS+ System (Bio-Rad Laboratories, Hercules, CA, USA) and the data were analyzed using Image J software (Version 1.50i, National Institute of Health, Bethesda, MD, USA).

### 3.7. Virtual Screening (HTVS)

A virtual screening process with α-amylase, GLUT2 and *h*-SGLT1 was carried out using two libraries of ligands’ compounds. These libraries were built taking into account the compounds found in the stigma and tepal extracts [20]. Pubchem and PhenolExplorer databases were consulted to retrieve the structures. To evaluate the binding mode and energy affinity of each compound inside the catalytic site of α-amylase enzyme and with the transporters, GLUT2 and *h-S*GLT1, receptor and ligand files coordinates were generated and obtained in a pdbqt file format. The calculations of electrostatical potential were performed using the GRID box dimension of 94 × 114 × 126 Å^3^ with 0.4 Å (grid space value) along XYZ axes for the *h-S*GLT1/GLUT2 receptors and of 98 × 90 × 98 Å^3^ with 0.3 Å (grid space value) for α-amylase. AutodockVina program was used to perform virtual screening based on a simple scoring function method.

### 3.8. Molecular Docking

Molecular docking was performed using Autodock 4.2.6 suite [75]. The calculation of the electrostatic potential was performed with the Autogrid4 tool, setting a grid box of (50 × 50 × 50) Å^3^ centered on the previously identified (form VS) ligands’ pose. The genetic algorithm (GA) was used for the pose generations of each ligand and the AMBER force-field-based scoring function was used for energy calculations as implemented in the docking software. The number of independent GA runs increased up to 100, and the grid spacing was kept at 0.375. The conformation analysis was performed using LGA (Lamarckian Genetic algorithm).

### 3.9. Molecular Dynamics

Two systems were built: α-amylase with catechin-3-o-gallate and α-amylase with sesamin, a flavonol and lignan compound, respectively. These ligands were chosen as they resulted the best-scoring compounds from the molecular docking and considering their dynamical behavior, we confirmed their binding stability. The complexes parametrization with CHARMM-GUI [76] solvate tool was performed using CHARMM36 forcefield [77], TIP3P model for water solvation including NaCl neutralizing physiological ion concentration (0.15 M). MD calculations were carried out on the best-scoring compounds for the two libraries using GROMACS 2020.6 [78]. After the model minimization, six equilibration phases and MD simulations were carried out. The overall time of each MD simulation was settled to 50 ns, with a time-step of 0.002 ps. Periodic boundary conditions (PBCs) were applied in all directions using a neighbor-searching grid type and setting at 1.4 nm, the cut-off distance for the short-range neighbor list. Electrostatic interactions were taken into account through implementing a fast and smooth particle-mesh Ewald algorithm with a 1.4 nm distance for the Coulomb cut-off.

### 3.10. Statistical Analysis

Wet-lab experiments were performed independently at least three times with duplicates/triplicates in each round. The results are reported as means ± S.D. unless otherwise stated. A one-way analysis of variance (ANOVA) was carried out with GraphPad PRISM 8.2 software. A *p* value of <0.05 was considered as significant. IC50 values related to inhibition of α-amylase activity and of glucose transport by extracts from stigmas and tepals were also obtained via a non-linear fit using the software GraphPad Prism 8.2.

## 4. Conclusions

Inhibition of glucose uptake in the small intestine may prevent hyperglycemia, a risk factor for diabetes. Our results indicate that the extracts from the floral parts of *C. sativus* which normally go to waste (tepals) exert in vitro inhibitory activity against α-amylase and interfere with glucose transporters, thus reducing intestinal glucose uptake as seen in Caco-2 cells. This inhibition was confirmed by the in silico study, which indicated physical interactions between TE and SE polyphenols and α-amylase and glucose transporters.

Overall, our data appear to demonstrate that TE and SE from *C. sativus* could potentially alleviate postprandial hyperglycemia through diminishing or regulating intestinal sugar absorption. The in vitro effect of TE and SE was observed at a concentration of polyphenols of about 20 µg GAE/mL (117 µmol GAE/L) (Table 2). It is worth bearing in mind that polyphenols are present in the stomach and intestinal lumen at millimolar concentrations after consumption of a diet rich in fruits and vegetables, whereas in plasma their concentration is much lower (<1 µmol/L) [79]. However, to determine the true efficacy and safety of both the short-term and long-term administration of TE and SE polyphenol-rich extracts, trials on human subjects for management of altered post-prandial glucose levels and metabolic syndrome are necessary. In this regard, in vivo studies are in progress to confirm the ability of tepal extracts to modulate post-prandial hyperglycemia. As such, it is also important to consider the interactions of these extracts within the gastrointestinal tract and how this may alter their inhibitory potential. Indeed, there is evidence that both catechins and procyanidins are stable in the acid environment of the stomach [80,81] and remain stable during intestinal transit [82]. However, pancreatic digestion, coupled with a shift to slightly alkaline pH, may cause degradation of the polymeric procyanidins to their respective monomeric components [81,82]. Further research should also focus on assessing the nature, isolation, purification and analysis of the individual polyphenols in the TE and SE extracts that are responsible for the positive effects reported here.

In the future, extracts from waste products of the saffron industry may be used to develop novel functional foods (bread, baked products) and beverages to prevent and manage type II diabetes, considering that the addition of such polyphenol-rich extracts should not affect texture, organoleptic nor nutritional properties in order for the enriched foods and beverages to be consumer-acceptable.

## Figures and Tables

**Table 1:** Total Polyphenol Content (TPC), Total Flavonoids Content (TFC) and antioxidant capacity measured using the ORAC assay of stigma (SE) and tepal (TE) extracts. The results are expressed as mean value ± S.D. (*n* = 3). * *p* < 0.001 vs. SE. (GAE = Gallic Acid Equivalents; CE = Catechin Equivalents; ORAC = Oxygen Radical Absorbance Capacity).

	SE	TE
TPC (mg GAE/g)	22 ± 4	35 ± 4 *
TFC (mg CE/g)	2.50 ± 0.30	5.40 ± 0.30 *
ORAC (mmol Trolox eq./g)	0.78 ± 0.11	1.68 ± 0.12 *

**Table 2:** α-Amylase inhibition by stigma (SE) and tepal (TE) extracts. The results are expressed as mean value ± S.D. (*n* = 3). * *p* < 0.001 vs. SE; ° *p* < 0.001 vs. acarbose.

Sample	IC50 (mg/mL)	IC50 (μg GAE/mL)
Acarbose	0.05 ± 0.07	-
SE TE	1.10 ± 0.08 0.60 ± 0.09 *^,^°	25.2 ± 3.3 21.5 ± 2.3 *

**Table 3:** Effect of stigma (SE) and tepal (TE) extracts on glucose absorption in Caco-2 differentiated cells. The results are expressed as mean value ± S.D. (*n* = 3). * *p* < 0.001 vs. SE; ° *p* < 0.001 vs. Phlorizin.

Sample	IC50 (mg/mL)	IC50 (μg GAE/mL)
Phlorizin	0.23 ± 0.01	-
SE TE	2.30 ± 0.02 ° 1.20 ± 0.02 *^,^°	53.09 ± 7.10 44.40 ± 6.20 *

**Table 4:** Binding energy values from virtual screening of best-scoring compounds present in the *C. sativus* stigmas library with α-amylase.

Stigma Compounds	Binding Free Energy (kcal/mol)
Sesamin	−10.1
Episesamin	−10.1
Carnosol	−9.30
Delphinidin-3-o-rutinoside	−9.20
Dihydroquercetin	−9.20
Cyanidin	−9.20
Carnosic acid	−9.10
Sesamolinol	−9.00
Luteolin	−8.80
Delphinidin-3-o-glucosylglucoside	−8.80
Medioresinol	−8.80

**Table 5:** Binding energy values from virtual screening of best scored compounds present in the *C. sativus* tepals library with α-amylase.

Tepal Compounds	Binding Free Energy (kcal/mol)
Epicatechin-3-o-gallate	−9.50
Catechin-3-o-gallate	−9.40
Cyanidin	−9.20
Quercetin-3-o-6-acetyl-galacatosyl-7-rhamnoside	−9.20
Kaempferol-3-o-acetylgalactosyl-7-o-rhamnoside	−9.10
Dihydroquercetin	−9.20
Epirosmanol	−9.20
Luteolin	−9.00
Rosmanol	−9.00
Scutellarein	−8.90
Delphinidin-3-o-glucosylglucoside	−8.90

**Table 6:** Binding energy values from virtual screening of best scored compounds present in the *C. sativus* stigmas library with GLUT2 receptor.

Stigma Compounds	Binding Free Energy (kcal/mol)
Episesamin	−8.60
Cyanidin-3,5-o-diglucoside	−8.60
Carnosol	−8.50
Delphinidin-3,5-o-diglucoside	−8.10
Cyanidin	−8.00

**Table 7:** Binding energy values from virtual screening of best scored compounds present in the *C. sativus* tepals library with GLUT2 receptor.

Tepal Compounds	Binding Free Energy (kcal/mol)
Delphinidin-3-o-glucosylglucoside	−8.80
Quercetin-3-o-acetylrhamnoside	−8.70
Epirosmanol	−8.50

**Table 8:** Binding energy values from virtual screening of best scored compounds present in the *C. sativus* stigmas library with h-SGLT1 glucose transporter.

Stigma Compounds	Binding Free Energy (kcal/mol)
Delphinidin-3,5-o-diglucoside	−9.1
Delphinidin-3-o-rutinoside	−8.5
Cyanidin	−8.0
Luteolin	−8.0
Episesamin	−8.3
Sesamolinol	−8.45

**Table 9:** Binding energy values from virtual screening of best scored compounds present in the *C. sativus* tepals library with h-SGLT1 glucose transporter.

Tepal Compounds	Binding Free Energy (kcal/mol)
Malvindin-3,5-o-diglucoside	−9.0
Delphinidin-3-o-glucoside	−9.1
Kaempferol-3-o-acetylglucoside	−8.5
Epicatechin-3-o-gallate	−8.5
Quercetin-3-o-acetylrhamnoside	−8.4
Scutellarein	−8.1

## Data Availability

The data presented in this study are available on request from the corresponding author.

## Supplementary Materials

The supporting information can be downloaded at: https://www.mdpi.com/article/10.3390/ijms24119213/s1.

## Abbreviations

BCA	Bicinchoninic Acid Assay
Caco2	Human colorectal adenocarcinoma cells
CBD	Carbohydrates binding domain
CE	Catechin equivalents
DMEM	Dulbecco’s modified Eagle’s medium
DPBS	Dulbecco’s phosphate-buffered saline
FBS	Fetal bovine serum
GA	Genetic algorithm
GAE	Gallic acid equivalents
GLUT2	Glucose transporter 2
GLUT3	Glucose transporter 3
HESI	Heated electrospray ionization interface
h-SGLT1	Human sodium glucose co-transporter-1
HTVS	High-throughput virtual screening
IC50	Half maximal inhibitory concentration
ICH	Intracellular helices
LGA	Lamarckian-genetic algorithm
MD	Molecular dynamics
ORAC	Oxygen radical absorbance capacity
PAHBAH	4-hydroxybenzhydrazide
PBCs	Periodic boundary conditions
PVDF	Polyvinylidene fluoride membrane
SDS-PAGE	Sodium dodecyl sulphate-polyacrylamide gel electrophoresis
SE	Stigma extracts
SGLT1	Sodium glucose co-transporter-1
SPE	Solid-phase extraction
T1D	Type 1 diabetes
T2D	Type 2 diabetes
TE	Tepal extracts
TEER	Transepithelial electrical resistance
TFC	Total flavonoids
TPC	Total polyphenols
U-HPLC-HRMS	Ultra-high performance liquid chromatography coupled to high-resolution mass spectrometry
