# Supplementary material for: Insights on the Hypoglycemic Potential of Crocus sativus Tepal Polyphenols: An In Vitro and In Silico Study
Source: Int J Mol Sci. 2023 May 24;24(11):9213. doi: 10.3390/ijms24119213 (PMC10252962; doi:10.3390/ijms24119213)
Supplement: Supplementary file 1 [file ijms-24-09213-s001.zip › Figure S3.pdf]

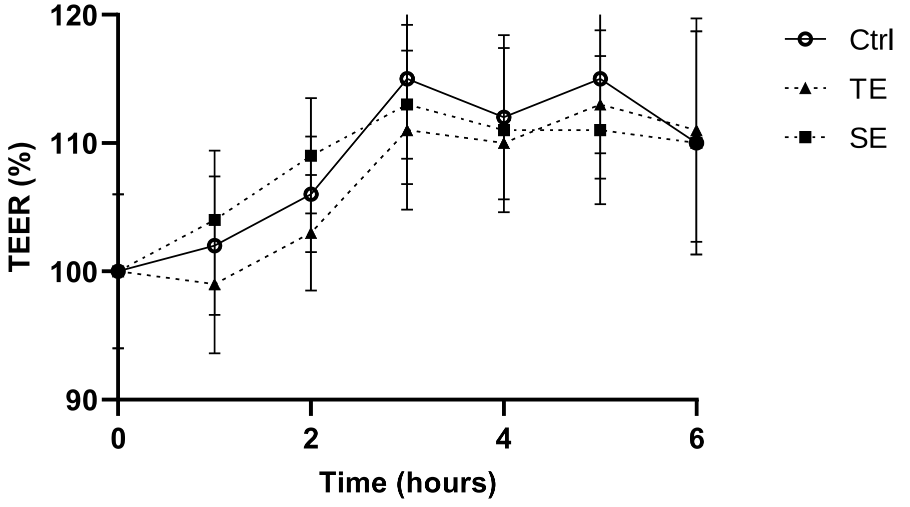

Figure S3: Transepithelial electrical resistance (TEER) across the differentiated monolayer of Caco-2 cells incubated in the absence (CTRL) or in the presence of tepal (TE) and stigma (SE) extracts. Differentiated Caco-2 cells were incubated for 6 h in the presence of 5 mM glucose (CTRL) and with TE or SE (50 μg GAE/mL).
