# Supplementary material for: Insights on the Hypoglycemic Potential of Crocus sativus Tepal Polyphenols: An In Vitro and In Silico Study
Source: Int J Mol Sci. 2023 May 24;24(11):9213. doi: 10.3390/ijms24119213 (PMC10252962; doi:10.3390/ijms24119213)
Supplement: Supplementary file 1 [file ijms-24-09213-s001.zip › Supplementary Table S2.pdf]

Table S2: Alignment between hglut2 and hglut3 sequences performed with ClustalOmega.

```
hglut2      MTEDKVTGTLVFTVITAVLGSFQFGYDIGVINAPQQVIISHYRHVLGVPLDDRKAINNYV      60
hglut3      MGTQKVTPALIFAITVATIGSFQFGYNTGVINAPEKIIKEFITKTLT----D--------      48
            *  :*** :*:*:: .*.:*******: ******:::* ..  :.*     *

hglut2      INSTDELPTISYSMNPKPTPWAEEETVAAAQLITMLWSLSVSSFAVGGMTASFFGGWLGD      120
hglut3      ----------------------KGNAPPSEVLLTSLWSLSVAIFSVGGMIGSFSVGLFVN      86
                                  : ::  :  *:* ******: *:**** .**  * : :

hglut2      TLGRIKAMLVANILSLVGALLMGFSKLGPSHILIIAGRSISGLYCGLISGLVPMYIGEIA      180
hglut3      RFGRRNSMLIVNLLAVTGGCFMGLCKVAKSVEMLILGRLVIGLFCGLCTGFVPMYIGEIS      146
             :** ::**:.*:*::.*. :**:.*:. *  ::* ** : **:*** :*:********:

hglut2      PTALRGALGTFHQLAIVTGILISQIIGLEFILGNYDLWHILLGLSGVRAILQSLLLFFCP      240
hglut3      PTALRGAFGTLNQLGIVVGILVAQIFGLEFILGSEELWPLLLGFTILPAILQSAALPFCP      206
            *******:**::**.**.***::**:*******. :** :***:: : *****  * ***

hglut2      ESPRYLYIKLDEEVKAKQSLKRLRGYDDVTKDINEMRKEREEASSEQKVSIIQLFTNSSY      300
hglut3      ESPRFLLINRKEEENAKQILQRLWGTQDVSQDIQEMKDESARMSQEKQVTVLELFRVSSY      266
            ****:* *: .** :*** *:** * :**::**:**:.*  . *.*::*::::**  ***

hglut2      RQPILVALMLHVAQQFSGINGIFYYSTSIFQTAGISKPVYATIGVGAVNMVFTAVSVFLV      360
hglut3      RQPIIISIVLQLSQQLSGINAVFYYSTGIFKDAGVQEPIYATIGAGVVNTIFTVVSLFLV      326
            ****:::::*:::**:****.:*****.**: **:.:*:*****.*.** :**.**:***

hglut2      EKAGRRSLFLIGMSGMFVCAIFMSVGLVLLNKFSWMSYVSMIAIFLFVSFFEIGPGPIPW      420
hglut3      ERAGRRTLHMIGLGGMAFCSTLMTVSLLLKDNYNGMSFVCIGAILVFVAFFEIGPGPIPW      386
            *:****:*.:**:.** .*: :*:*.*:* :::. **:*.: **::**:***********

hglut2      FMVAEFFSQGPRPAALAIAAFSNWTCNFIVALCFQYIADFCGPYVFFLFAGVLLAFTLFT      480
hglut3      FIVAELFSQGPRPAAMAVAGCSNWTSNFLVGLLFPSAAHYLGAYVFIIFTGFLITFLAFT      446
            *:***:*********:*:*. ****.**:*.* *   *.: * ***::*:*.*::*  **

hglut2      FFKVPETKGKSFEEIAAEFQKKSGSAHRPKAAVEM--KFLGATETV----  524
hglut3      FFKVPETRGRTFEDITRAFEGQAHGADRSGKDGVMEMNSIEPAKETTTNV  496
            *******:*::**:*:  *: :: .*.*      *  : :  :: .
```
