# Supplementary figures and images for: Insights on the Hypoglycemic Potential of Crocus sativus Tepal Polyphenols: An In Vitro and In Silico Study
Source: Int J Mol Sci. 2023 May 24;24(11):9213. doi: 10.3390/ijms24119213 (PMC10252962; doi:10.3390/ijms24119213)

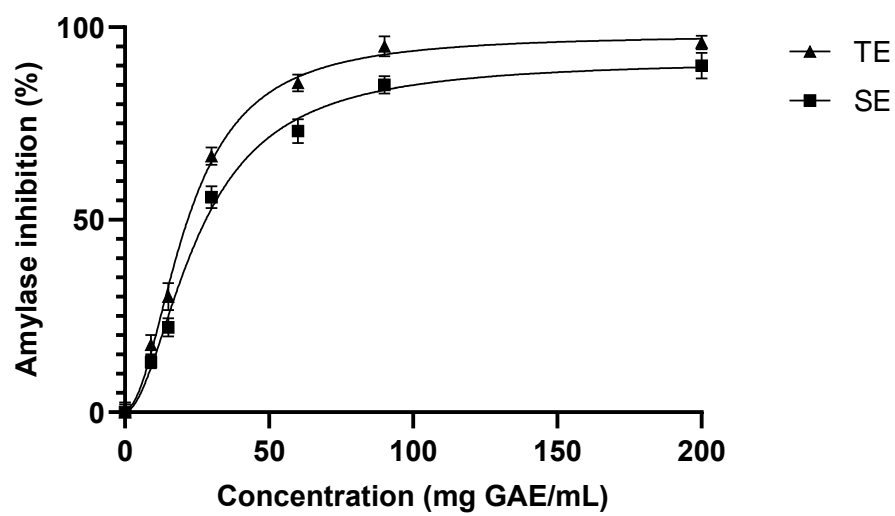

Figure S1:  $\alpha$ -Amylase inhibition curves of stigma (SE) and tepal (TE) extracts.

Supplement: Supplementary file 1 [file ijms-24-09213-s001.zip › Figure S1.pdf]
